# Dissolved organic matter protects mosquito larvae from damaging solar UV radiation

**DOI:** 10.1371/journal.pone.0240261

**Published:** 2020-10-07

**Authors:** Nicole L. Berry, Erin P. Overholt, Thomas J. Fisher, Craig E. Williamson

**Affiliations:** 1 Department of Biology, Miami University, Oxford, Ohio, United States of America; 2 Department of Statistics, Miami University, Oxford, Ohio, United States of America; University of Siena, ITALY

## Abstract

Mosquitoes have increased in their abundance and geographic distribution in northeastern North America, coinciding with an increase in extreme precipitation events and up to a doubling of dissolved organic matter (DOM) concentrations in some inland waters. Increases in DOM can reduce exposure of mosquito larvae to solar ultraviolet (UV) radiation. Although mosquito larvae are most common in shaded habitats, almost nothing is known about their susceptibility to damage by solar UV radiation, or the ability of DOM to create a refuge from damaging UV in their shallow-water habitats. We hypothesize that 1) exposure to solar UV radiation is lethal to mosquito larvae, 2) larvae lack photo-enzymatic repair to fix UV-damaged DNA, and 3) DOM shades larvae from lethal solar UV radiation. We tested these hypotheses with experiments that manipulated UV radiation, the photo-repair radiation necessary for photo-enzymatic DNA repair, and DOM. Exposure to solar UV radiation significantly decreased larval survivorship, while DOM significantly increased it. There was no evidence of photo-enzymatic DNA repair. Our findings confirm that solar UV radiation decreases habitat suitability for mosquito larvae, but DOM provides a refuge from UV. This highlights the need for vector control managers to prioritize high DOM and shaded habitats in their efforts to reduce mosquito populations.

## Introduction

Many mosquitoes, such as the invasive *Aedes aegypti* and *A*. *albopictus*, are vectors of diseases (i.e. Zika virus and dengue fever) and are expanding their habitat range to more northern latitudes of the continental United States of America [1–3]. In northeastern North America (NENA), this range expansion is correlated with heavier precipitation and warmer air temperatures [1–5], as well as with human-facilitated dispersal [6]. Additionally, the increased abundance of *Culex pipiens* and *C*. *restuans* in NENA forests has led to increased transmission of West Nile Virus to wildlife such as the ruffed grouse (*Bonasa umbellus*) [7]. The increase in mosquitoes and risk of disease transmission [8] requires a thorough understanding of the environmental variables regulating these disease vectors.

Current management strategies are challenged by the inability to selectively kill adult mosquitoes without killing beneficial insects [9], and by the fact that mosquitoes are rapidly evolving resistance to pesticides [5]. Furthermore, many species of mosquitoes are well adapted to survive in both artificial and naturally occurring water bodies [10], making the ability to selectively target the most critical habitats important for efficient management practices. Due to the potential human health implications for the spread of mosquitoes in urban areas, artificial container ecology of these mosquitoes has been the focus of the literature, and therefore may increase the chance of failure to detect and manage species in predominantly naturally occurring water bodies [11].

Along with the observed increases in mosquitoes and their associated diseases across NENA, there has been up to a doubling of the concentrations of dissolved organic matter (DOM, the terrestrially-derived leachate from leaves and other organic matter that passes through a filter) in many naturally occurring inland waters due to recovery from acid deposition, increased precipitation, and a variety of other factors [12–14]. Many mosquito larvae are commonly found in habitats that are physically shaded by plant cover [15, 16], and have high DOM concentrations, which are hypothesized to enhance food resources through a stimulation of the microbial food web [11, 17, 18]. DOM could provide another benefit to mosquitoes by shading their larvae from potentially damaging solar UV radiation. DOM selectively absorbs short wavelengths of radiation, such as UV-B radiation [19] which is known to include the most damaging wavelengths of light reaching the Earth’s surface [20]. Therefore, the selective absorption of UV-B radiation is known to provide a refuge from exposure to UV for many aquatic organisms that have a low UV-tolerance such as the predatory aquatic larvae of the phantom midge [21], zooplankton [22], and juvenile fish [23].

Mosquitoes have the potential to be damaged by UV radiation as demonstrated by their decreased survival after exposure to UV-C radiation from artificial sources such as UV-lamps [24]. However, UV-C radiation is not present in sunlight reaching Earth’s surface due to ozone and other pollutants blocking these wavelengths of radiation [25] and therefore UV-C is not a very realistic representation of the role that solar UV radiation may play in regulating mosquito populations. Currently, the UV-tolerance of mosquito larvae and the potential mechanisms of protection from damage by solar UV radiation are largely unknown.

In addition to DOM being a potential mediator of exposure to damaging UV radiation, molecular repair of UV-damaged DNA through photo-enzymatic repair (PER) may also reduce the negative effects of exposure to UV radiation. Longer wavelength UV-A radiation and visible light, collectively called photo-repair radiation (PRR), are required to stimulate photolyase—the enzyme critical for PER [22, 26]. However, short wavelengths of visible light have been shown to decrease survival of mosquito larvae [27]. These findings not only suggest that larvae are not capable of PER, but also highlight the potential importance of DOM in providing a refuge from damaging sunlight. No peer-reviewed published information is available on the effects of natural solar UV radiation on mosquito larvae, or whether larvae have the ability to repair UV-damaged DNA through PER.

Here we test three hypotheses: (1) natural solar UV radiation kills mosquito larvae, (2) mosquito larvae do not have PER capabilities, and (3) DOM increases survivorship of mosquito larvae exposed to UV radiation. Testing these hypotheses has the potential to create new insights into the consequences of increases in DOM on the observed expansion of mosquitoes and their associated diseases across NENA, with important implications for mosquito management.

## Materials and methods

*C*. *pipiens* and *C*. *restuans* mosquitoes are two mosquitoes common across NENA that are found in both artificial and natural water bodies and are known vectors of detrimental diseases such as West Nile Virus—therefore making them suitable model species of mosquitoes to test our hypotheses [28]. Both species were collected as egg rafts from ovipositing traps located in forested areas around either Lacawac Sanctuary Biological Field and Research Station (Wayne County, Pennsylvania, USA, 41.376868, -75.300318) or the Ecology Research Center (ERC) at Miami University (Butler County, Ohio, USA, 39.531830, -84.723106) and then hatched in the laboratory (S1A Fig in S1 File). The Ecology Research Center is part of Miami University and does not require additional permissions. The use of Lacawac Sanctuary for collecting egg rafts and conducting experiments was permitted on May 22, 2018 by the Director of Science and Research, Dr. Beth Norman and Lacawac Sanctuary’s Science Committee. In cases where multiple egg rafts were used, all larvae were homogenously mixed into a container before being used for an experiment, and a subsample of larvae were removed from each egg raft for identification. First instar larvae were exposed to various light manipulations using both natural (“solar”) and artificial (“UV-lamp”) sources of radiation in experimental setups called “phototrons” [22]. All phototron treatments used at least ten replicate dishes with at least five larvae per replicate dish and lasted for 12 hours. After the 12-hour exposure, mosquito larvae were scored for survivorship (alive or dead) and the living larvae were transferred to a holding dish of -DOM water with excess food and incubated at 26°C in a dark environmental chamber. The presence of DOM has the potential to have confounding effects on the growth and development of the larvae due to the increase in food. Therefore, only survivorship immediately following the 12-hour exposure period was used as the end point. The potential for sublethal effects during this time period likely make this a conservative estimate of the negative effects of UV on morbidity and mortality.

### Description of solar phototron design

A solar phototron experiment was conducted under natural solar radiation at Lacawac Sanctuary on July 13^th^, 2018 on a partly cloudy day, beginning shortly after sunrise (6:15am) and ending a few hours before sunset (6:15pm). Our modified version of the solar phototron originally described in Williamson et al. (2001) [22] accommodated 164 dishes suspended by 2.54 cm-thick housing insulation which floated on top of a leveled 100 cm x 28 cm pool of water (S1B Fig in S1 File). A constant inflow of cold water into the pool maintained the water temperature inside the phototron dishes below 35°C to avoid overheating. Average temperatures were 24.9 and 24.7°C in the presence and absence of solar UV radiation, respectively. Solar UV radiation was manipulated with special light-filtering plastic covers placed on top of the dishes that either reduced UV radiation with Courtgard (CP Films Inc., Martinsville, VA, USA; ~87% transmittance of 400–700 nm PAR, 6% of 320–399 nm UV-A, and 0% of 295–319 nm UV-B radiation) or transmitted UV radiation with Aclar (Honeywell International, Pottsville, PA, USA; ~93% transmittance of 400–700 nm PAR, 92% of 320–399 nm UV-A, and 90% of 295–319 nm UV-B [29]). A portable UV radiometer (BIC IL, Biospherical Instruments, Inc., San Diego, CA) recorded the day’s exposure of 6.11 kJm^-2^nm^-1^ ambient solar UV radiation at 320nm. This is approximately 56% of maximum 320nm exposure at 41°N during summer solstice on a cloud-free day (10.9 kJm^-2^nm^-1^, [30]).

### Description of UV-lamp phototron design

For UV-lamp phototrons, which were conducted under artificial UV conditions in the laboratory, larvae were placed into 40 quartz dishes (100% UV transmittance) and rotated horizontally below one UV-B lamp and above four fluorescent PRR lamps (two 40 W cool-white and two UV-A bulbs providing PRR from below the dishes) as described in Williamson et al. (2001) [22]. Multiple phototron trials were conducted for 12 hours at a time and temperatures were kept at 26 ± 2°C. Variation between trials was reduced by conducting two by two full factorial designs, always manipulating the presence of UV-B radiation (*H1*), and either PRR (*H2*) or DOM (*H3*). To block exposure to UV-B or PRR, a solid black PVC lid was placed either on top (for–UV-B exposure) or on the bottom (for–PRR exposure) of the dishes. For more specifics regarding the spectral composition of the PRR and UV-B lamps see Williamson et al. (2001) [22].

### DOM water preparation and analyses

DOM stock was prepared by adding 800 g leaf litter (primarily dried oak leaves from the forest floor) to 10 L of tap water and letting the mix soak for a minimum of 48 hrs. After soaking, water was strained through a series of sieves down to a 48 μm sieve and tap water was used to make a 1:10 dilution of this stock solution. Dissolved organic carbon (DOC) concentrations were determined by filtering both DOM (+DOM treatment) and tap water (-DOM treatment) using a pre-ashed Whatman GF/F (0.7 μm) filter. Samples were analyzed using a Total Organic Carbon Analyzer (TOC-V_CPH,_ Shimadzu), and DOC concentrations were calculated by subtracting a DI blank and calibrating values using a certified standard. Absorbance was measured using a spectrophotometer (UV/Visible 1650-PC, Shimadzu) and a Naperian dissolved absorption coefficient was calculated as described in Williamson et al. (2015) [31]. DOC concentrations were 0.6 mg C L^-1^ (-DOM) and 18.3 mg C L^-1^ (+DOM). The 320nm absorption coefficients were 1.1 nm^-1^ (-DOM) and 49.2 nm^-1^ (+DOM). The DOM treatment values are comparable to a nearby vernal pool where adult mosquitoes are found (11.1 mg C L^-1^, 40.3 nm^-1^). Both types of water were allowed to sit out for a minimum of 48 hours before being used in the experiments.

### Mosquito survivorship statistical analyses

A generalized linear mixed effects model with a binomial response was fit on 219 dishes of larval survival as a function of eight distinct treatments, as follows:

$$\log(\frac{p}{1-p})=\gamma +\sum_{i=1}^{8}\beta_{i}*X_{i}$$

where *γ* was a random effect on each replicate dish within a treatment (thus controlling for chamber effects within each phototron dish; [32]), *β*_*i*_ was the effect of each of the distinct phototron treatments, *X*_*i*_ was an indicator variable (1 or 0) determining the distinct treatments, and *p* was the proportion of mosquito larvae surviving. The incorporation of the dish as a random effect in the model accounts for any potential influence an individual larva may have on another larva within an individual replicate dish given the variability in the number of individual larvae per replicate dish.

The overall model was determined to be significant to predict survivorship by a likelihood ratio test (Chi-sq: 229.7, *P-value = 2*.*20e-16*). Statistical tests to assess our hypotheses consisted of 10 comparisons and a Bonferroni correction family-wise error rate was applied (for 95% confidence interval we considered *P-value <0*.*005* as statistically significant, and *P-values* between 0.005–0.05 were considered marginally significant and interpreted as having suggestive effects).

Statistical modeling of treatments with either 0% or 100% survivorship in all replicate dishes cannot estimate standard errors and therefore makes statistical hypothesis testing untenable. In the case of 0% survivorship, the number of larvae that survived in a dish was randomly reassigned to 0 (0.80 probability) or 1 (0.20 probability). In the case of 100% survivorship, the number of surviving larvae in a dish was randomly reassigned from *n* to *n-1* with probability 0.20, where there were *n* larvae in a dish. This modification does not change the results of the experiment but improves the model fitting accuracy and our model agrees with the exploratory data analysis.

All data can be found in the supplemental information (S1 Dataset). All analyses used R Project for Statistical Computing [33] through the lme4 package [34] for the model building and interpretation and tidyverse package [35] for data cleaning and figures.

## Results

**Fig 1:**
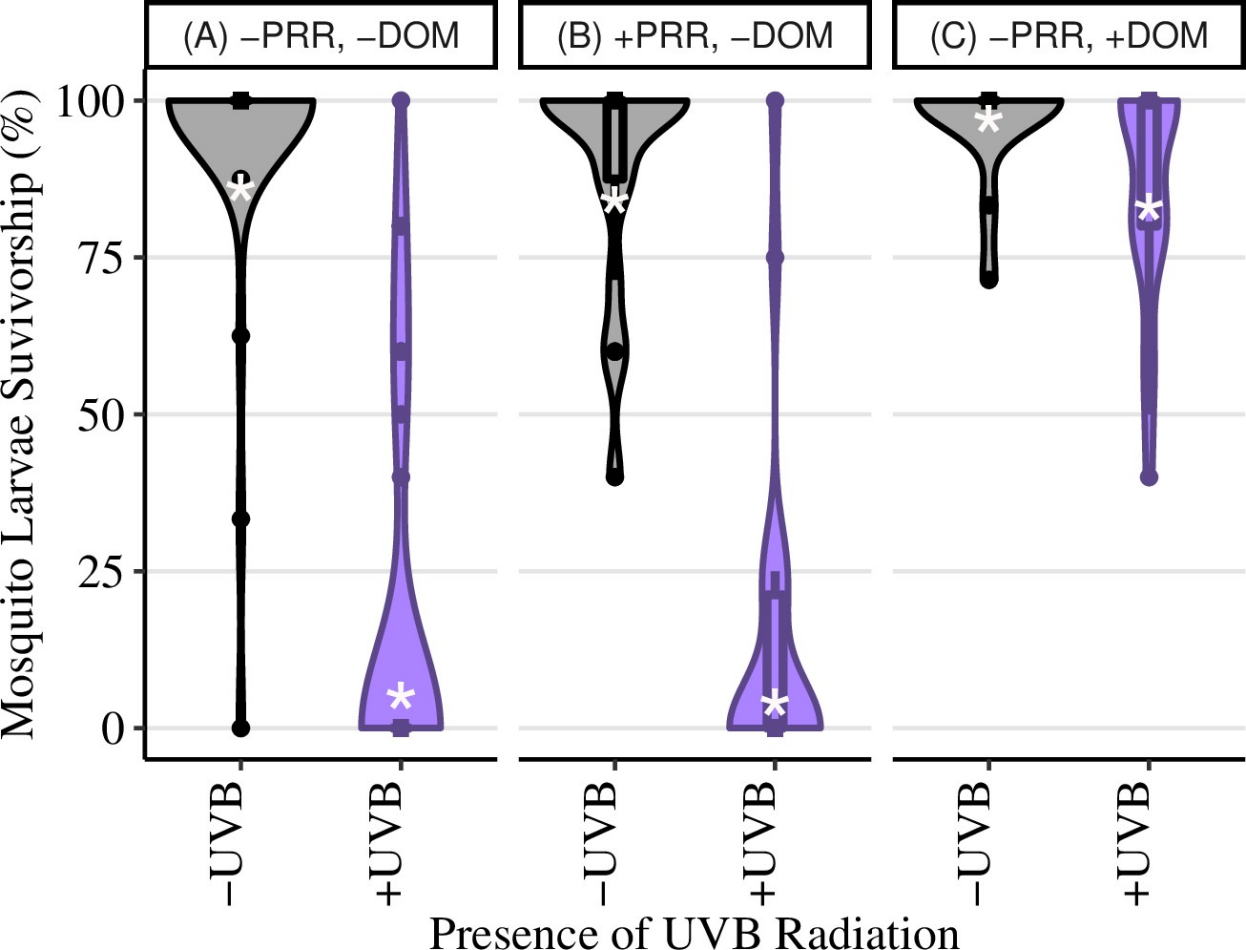
Solar phototron survivorship. Mosquito percent survivorship in the solar (field) phototron experiment conducted at the field at Lacawac Sanctuary, (PA), with ambient UV radiation exposure of 6.11 kJm^-2^nm^-1^ using first instar *C*. *restuans* larvae. Only the presence of UV radiation was manipulated, with data represented as violin plots. Violin plots use the width of plot represents the relative number of replicates with that percent survivorship. White asterisks represent the predicted percent survival from the statistical model. Box and whiskers plots were overlaid onto the violin plots to display the distribution of percent survivorship in each dish. Lower and upper hinges represent the 1^st^ and 3^rd^ quartiles with whiskers determined by the smallest and largest values 1.5 times the interquartile range of the hinges, and individual points represent outliers.

**Fig 2:**
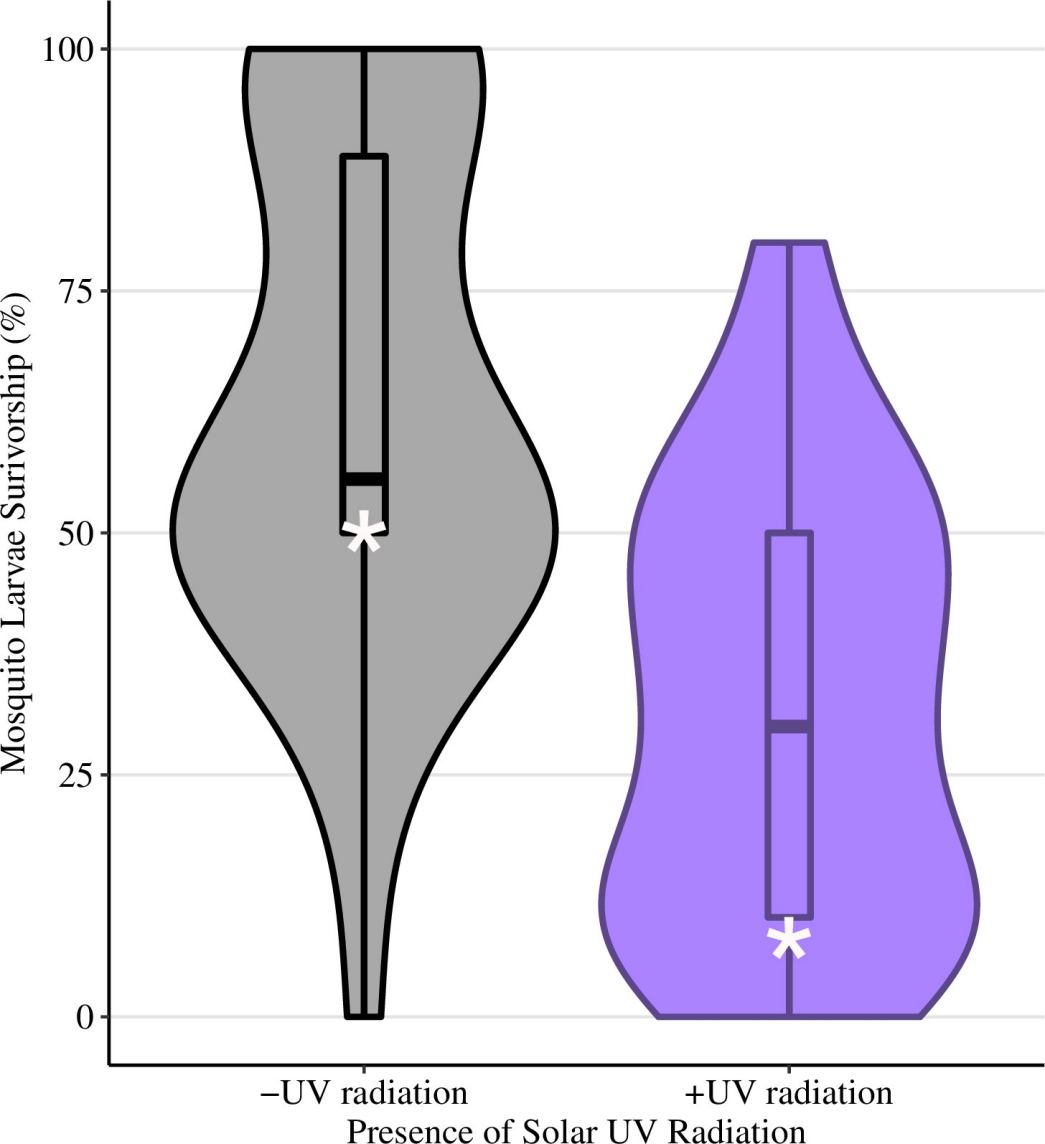
UV-lamp phototron survivorship. Mosquito percent survivorship data collected from the UV-lamp (laboratory) phototrons using a mix of first instar larval *C*. *pipiens* and *C*. *restuans*. UV-B radiation was manipulated in the absence of DOM and PRR **(A)**, in the presence of PRR **(B)**, and in the presence of DOM **(C)**. Violin plots use the width of plot represents the relative number of replicates with that percent survivorship. White asterisks represent the predicted percent survival from the statistical model. Box and whiskers plots were overlaid onto the violin plots to display the distribution of percent survivorship in each dish. Lower and upper hinges represent the 1^st^ and 3^rd^ quartiles with whiskers determined by the smallest and largest values 1.5 times the interquartile range of the hinges, and individual points represent outliers.

Overall the statistical model and corresponding comparisons demonstrated that the presence of solar UV radiation decreased survivorship, and there was no evidence that PER significantly increased survival, however, the presence of DOM did (Table 1). Even at solar UV exposure levels of only about half of the maximum predicted for this region on summer solstice, the presence of solar UV radiation decreased median survivorship from 56% to 30% (Fig 1). The presence of UV-B radiation from the UV-lamp decreased median survivorship from 100% to 0% (Fig 2). The presence of PRR had no effect on median survival during exposure to UV-B lamp radiation (0% for both treatments; Fig 2), indicating that PER, if present, was not strong enough to overcome damage by UVB radiation. In the absence of UV-B lamp radiation, larvae exposed to only PRR had 100% median survivorship (Fig 2), indicating these relatively low levels of PRR (compared to the intensity of natural solar radiation [22]) were not damaging to mosquito larvae. When larvae were exposed to UV-B lamp radiation in the presence of DOM, survivorship was 100% (Fig 2).

**Table 1:** Results of the mixed effects logistic model for solar and UV-lamp phototrons. Results of the mixed effects logistic model for each of the 10 statistical comparisons made within each phototron. Relative percent survivorship was calculated by subtracting the estimated percent survivorship of treatment B from treatment A (i.e. a positive value indicates that treatment B resulted in lower survivorship than treatment A as predicted by the statistical model).

Phototron Fixed Treatments Compared (Treatment A)—(Treatment B)	Significance (*P-value*)	Relative Difference in Estimated Percent Survival
Solar Phototron Comparison (field):		
**(-UV)—(+UV)**	**3.56e-05**	**42%**
UV-Lamp Phototron Comparisons (laboratory):		
**(-UVB, -PRR, -DOM)—(+UVB, -PRR, -DOM)**	**<2.00e-16**	**81%**
(-UVB, -PRR, -DOM)—(-UVB, +PRR, -DOM)	0.158	3%
**(-UVB, -PRR, -DOM)—(+UVB, +PRR, -DOM)**	**<2.00e-16**	**82%**
(-UVB, -PRR, -DOM)—(-UVB, -PRR, +DOM)	0.916	-10%
*(-UVB*, *-PRR*, *-DOM)—(+UVB*, *-PRR*, *+DOM)*	*0*.*033*	*3%*
(+UVB, -PRR, -DOM)—(+UVB, +PRR, -DOM)	0.709	1%
**(+UVB, -PRR, -DOM)—(+UVB, -PRR, +DOM)**	**1.18e-15**	**-78%**
**(-UVB, +PRR, -DOM)—(+UVB, +PRR, -DOM)**	**2.27e-13**	**80%**
(-UVB, -PRR, +DOM)—(+UVB, -PRR, +DOM)	0.098	14%

Significant differences (determined by *P-values* < 0.005) are bolded while marginally significant differences are italicized.

## Discussion

The hypotheses that both natural solar UV radiation and artificial UV-B radiation decrease larval survivorship were supported. There was no evidence that PER acted as an effective mechanism for overcoming the UV exposure levels by successfully increasing mosquito larvae survivorship. In part, this could be because the UV-lamp emits substantially lower levels of PRR than what is in natural sunlight [22], and consequently the survivorship in the PRR only treatment in the solar phototron was much lower than that observed in the PRR only treatment of the UV-lamp phototron. This is consistent with the findings of Hori et al. (2014) [27] who found that high levels of artificial blue light kill mosquito larvae. While further studies quantifying DNA damage and the potential for PER are required to better understand the potential for PER, our study supports the role of DOM providing a refuge in highly transparent waterbodies.

In addition to PER, other organism-level responses that could potentially reduce damage by solar UV radiation include nucleotide excision (dark) repair (NER), behavioral avoidance, and the presence of photo-protective compounds [22, 36, 37]. This study did not explicitly quantify DNA damage and repair to make any inferences regarding the efficiency of dark DNA repair mechanisms such as NER, which is a general DNA repair process that many organisms are thought to have [38]. Early instar larvae are restricted to air-water interfaces due to their breathing siphon—suggesting limited ability to behaviorally avoid UV radiation [39]. These larvae are also very transparent, suggesting a lack of visible photoprotective compounds, although mosquitoes have not been tested for less visible, UV-absorbing photoprotective compounds such as mycosporine-like amino acids, or antioxidant enzymes that provide protection from damaging UV radiation [40–42]. Future work needs to include further investigations of these other organism-level mechanisms of protection to better understand the necessity of a DOM refuge in larval mosquito habitats.

Other habitat characteristics of mosquitoes are changing and likely to play an important role in increases in mosquito abundance as well. Increases in temperature can increase adult survival [3, 4]; however, the consequences of changes in water temperature from different sources of shading on mosquito larval survival and development is less understood. For instance, the dark color of DOM absorbs sunlight and increases water temperatures, while shading by riparian tree cover will reduce incident sunlight and decrease water temperatures. We propose that while both types of shading may provide sufficient protection from lethal solar UV radiation, the consequences of these differences in water temperature on larval survivorship and growth rates, as well as on adult oviposition and fecundity need to be further investigated.

Additionally, it is well known that the source of DOM can play an important role in the absorption of UV radiation [13, 43]. Our study used terrestrially derived (allochthonous) sources of DOM, which are known to have more chromophoric properties than autochnothous sources of DOM and are the dominant source of DOM in north temperature lakes [44]. It is likely that many artificial water bodies that mosquito larvae are abundantly found have primarily allochthonous sources of organic matter from debris (i.e. leaf litter and detritus) falling into the containers. A follow-up study investigating the influence of the source of DOM on mosquito larvae survivorship from exposure to damaging UV radiation and other indirect effects (i.e. food subsidy for the microbial food web) is needed.

Regional decreases in acid deposition in NENA since the 1990’s is leading to increases in DOM concentrations across many inland water bodies. Despite the potential for solar UV radiation to act as a natural disinfectant for aquatic ecosystems [45], few studies have investigated the role of browning in increasing the prevalence of aquatic-borne diseases—with even fewer studies investigating the consequences of browning on aquatic-borne disease vectors such as mosquitoes. This is the first study to demonstrate that direct exposure to natural, solar UV radiation can decrease the survivorship of mosquito larvae, and to report that DOM can mitigate damage by UV-B radiation. Managers should consider the presence of natural sunlight when treating mosquito larval habitats. Currently, the literature has focused on studying the efficacy of larvicides in high UV environments [46, 47]. However, efforts on establishing the UV-tolerance of these larvicides should incorporate the UV-tolerance of mosquito larvae, given our study suggests that in high UV environments, exposure to the natural sunlight may be enough to effectively kill the first instar stage of these larvae. Alternatively, underground drainage systems which have limited exposure to sunlight and often have standing bodies of water, could serve as optimal breeding habitats for mosquitoes [48]. These waterbodies would not be capable of using natural sunlight to eliminate mosquito larvae, but UV-intolerant larvicides would be more effective. Alternatively, governing agencies may consider efforts to increase water transparency and exposure to natural sunlight in stormwater drainage systems, to reduce mosquito breeding habitats given our findings. More locally, homeowners with small bird baths or ponds located around the property may consider regularly emptying leaves or other organic debris found within the water to increase the potential for disinfection by solar UV radiation rather than applying chemicals to the water. Our findings argue for the consideration of exposure to natural sunlight to optimize treatment effectiveness.

More broadly, this research provides novel insight to addressing one of the National Research Council’s grand challenges in environmental sciences which includes a call for development of new approaches for surveillance and monitoring of the spread of invasive species and infectious diseases and their disease vectors [49]. In response to this call, NEON has since published two monitoring programs geared towards addressing the spread of infectious diseases by vectors with mosquitoes as a “sentinel taxon” [50, 51]. In both survey designs, the focus is on the adult stage using CDC CO_2_ bait traps across multiple land use-types based on the National Land Cover database [50, 51]. These surveys will help indicate where mosquitoes are most abundant, and if coupled with the findings of our research, could enable managers to focus their treatment efforts on habitats that are well-shaded and high in DOM concentrations.

Across NENA, invasive *Aedes* species mosquitoes are encroaching while native mosquito abundances continue to rise along with the prevalence of diseases such as EEE and West Nile virus. Our research provides a direct link between UV as a natural environmental regulator for these disease vectors and DOM, which is increasing in many parts of the world, and may be providing additional habitat for mosquito larvae.

## Supporting information

S1 File File containing supplemental images S1A Fig and S1B Fig. (DOCX)

S1 Dataset Datafile for data analysis. (XLS)
