# Correction: Dissolved organic matter protects mosquito larvae from damaging solar UV radiation

**DOI:** 10.1371/journal.pone.0244832

**Published:** 2020-12-28

**Authors:** Nicole L. Berry, Erin P. Overholt, Thomas J. Fisher, Craig E. Williamson

The images for Figs 1 and 2 are incorrectly switched. The image that appears as Fig 1 should be Fig 2, and the image that appears as Fig 2 should be Fig 1. The figure captions appear in the correct order.

**Fig 1:**
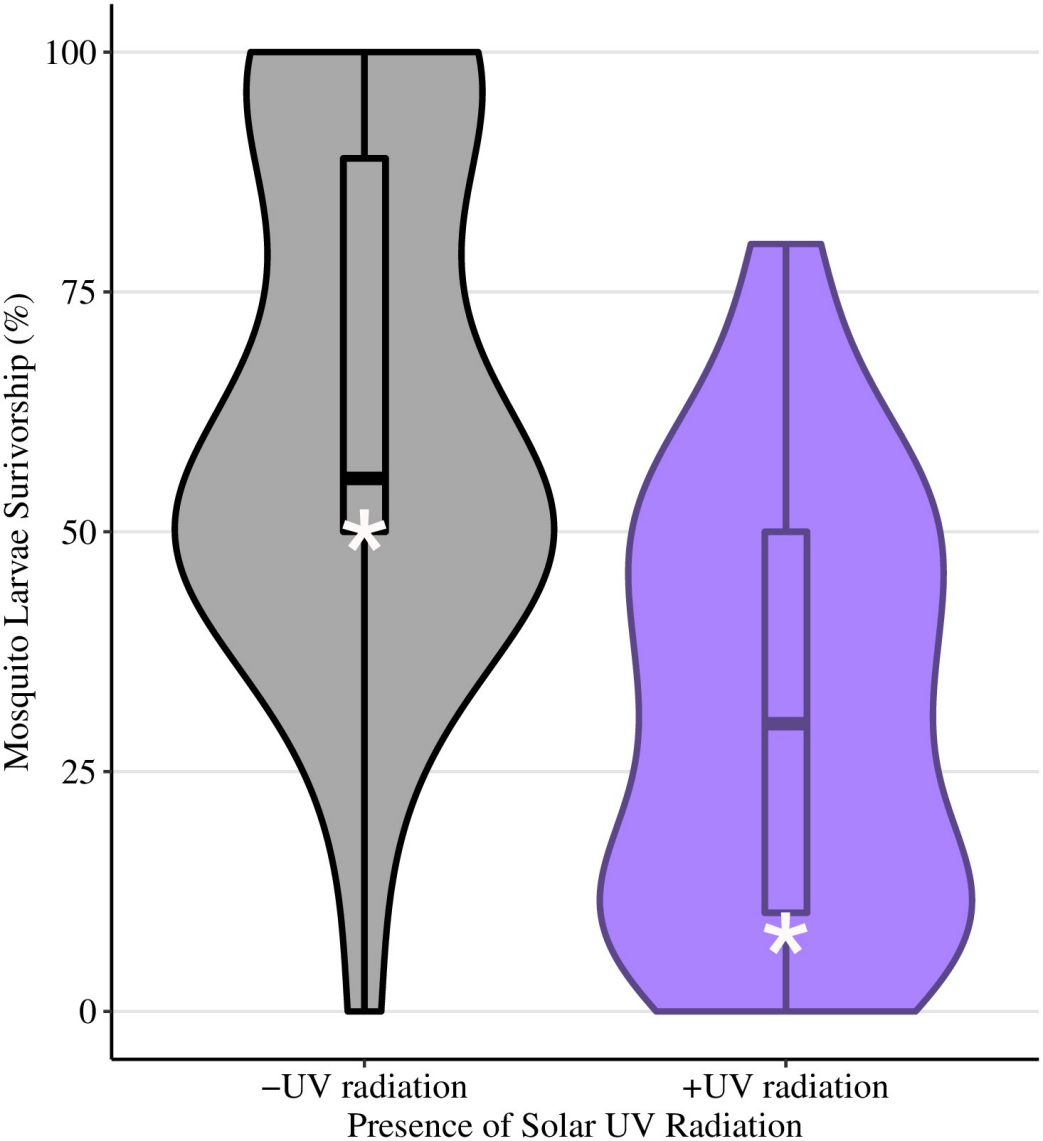
Solar phototron survivorship. Mosquito percent survivorship in the solar (field) phototron experiment conducted at the field at Lacawac Sanctuary, (PA), with ambient UV radiation exposure of 6.11 kJm^-2^nm^-1^ using first instar C. *restuans* larvae. Only the presence of UV radiation was manipulated, with data represented as violin plots. Violin plots use the width of plot represents the relative number of replicates with that percent survivorship. White asterisks represent the predicted percent survival from the statistical model. Box and whiskers plots were overlaid onto the violin plots to display the distribution of percent survivorship in each dish. Lower and upper hinges represent the 1^st^ and 3^rd^ quartiles with whiskers determined by the smallest and largest values 1.5 times the interquartile range of the hinges, and individual points represent outliers.

**Fig 2:**
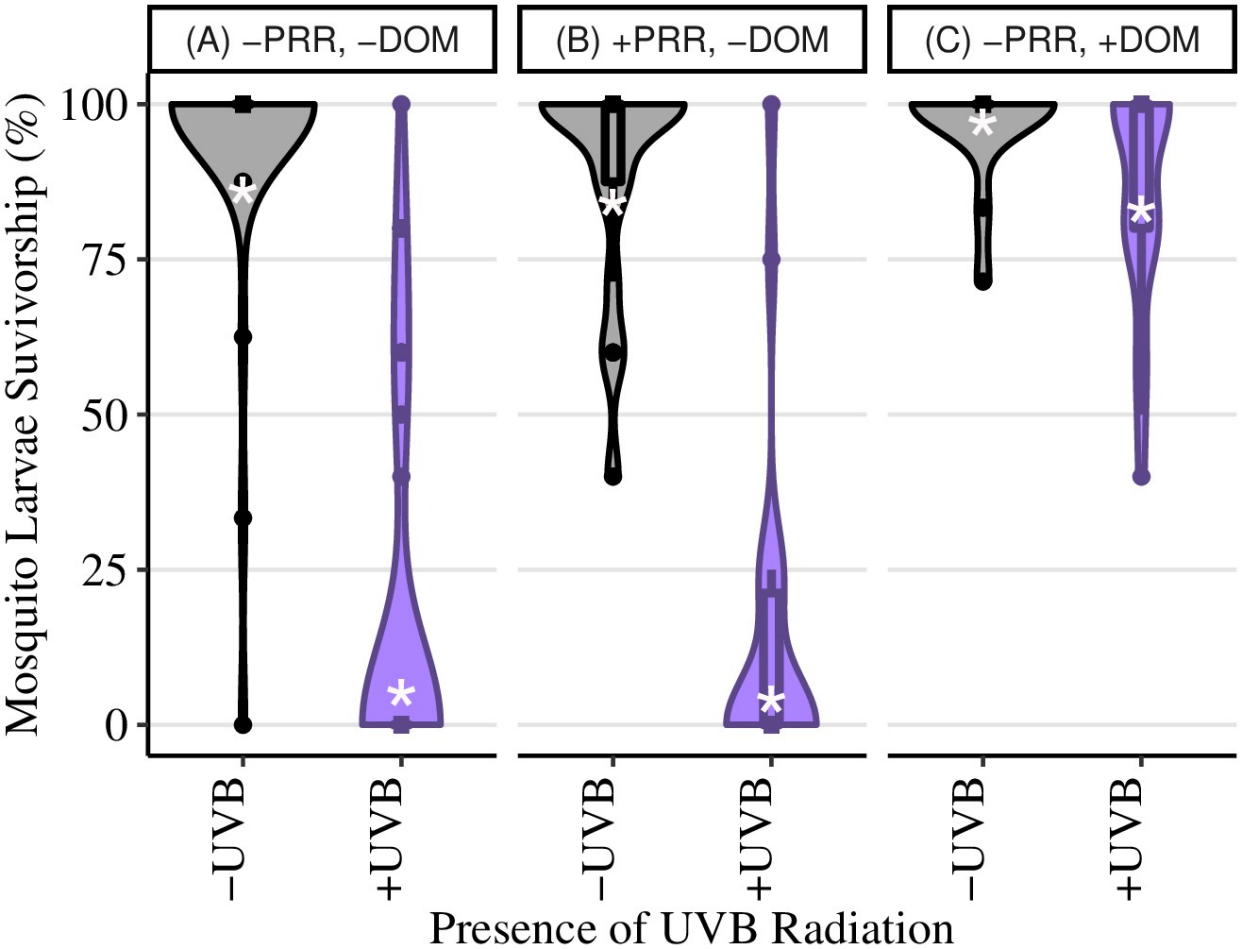
UV-lamp phototron survivorship. Mosquito percent survivorship data collected from the UV-lamp (laboratory) phototrons using a mix of first instar larval C. *pipiens* and C. *restuans*. UV-B radiation was manipulated in the absence of DOM and PRR **(A)**, in the presence of PRR **(B)**, and in the presence of DOM **(C)**. Violin plots use the width of plot represents the relative number of replicates with that percent survivorship. White asterisks represent the predicted percent survival from the statistical model. Box and whiskers plots were overlaid onto the violin plots to display the distribution of percent survivorship in each dish. Lower and upper hinges represent the 1^st^ and 3^rd^ quartiles with whiskers determined by the smallest and largest values 1.5 times the interquartile range of the hinges, and individual points represent outliers.
