# Supplementary material for: Dissolved organic matter protects mosquito larvae from damaging solar UV radiation
Source: PLoS One. 2020 Oct 7;15(10):e0240261. doi: 10.1371/journal.pone.0240261 (PMC7540860; doi:10.1371/journal.pone.0240261)
Supplement: S1 File — (DOCX) [file pone.0240261.s001.docx]

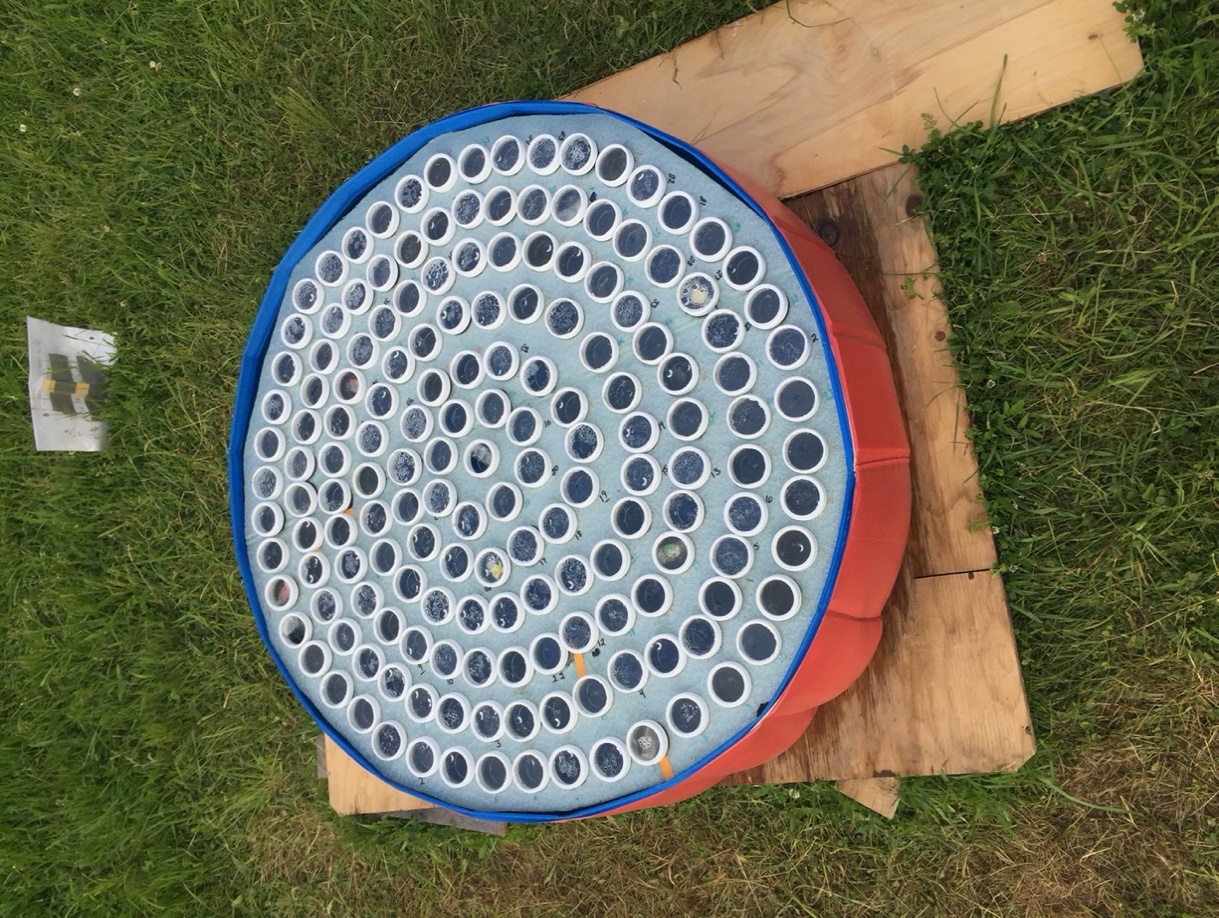
**
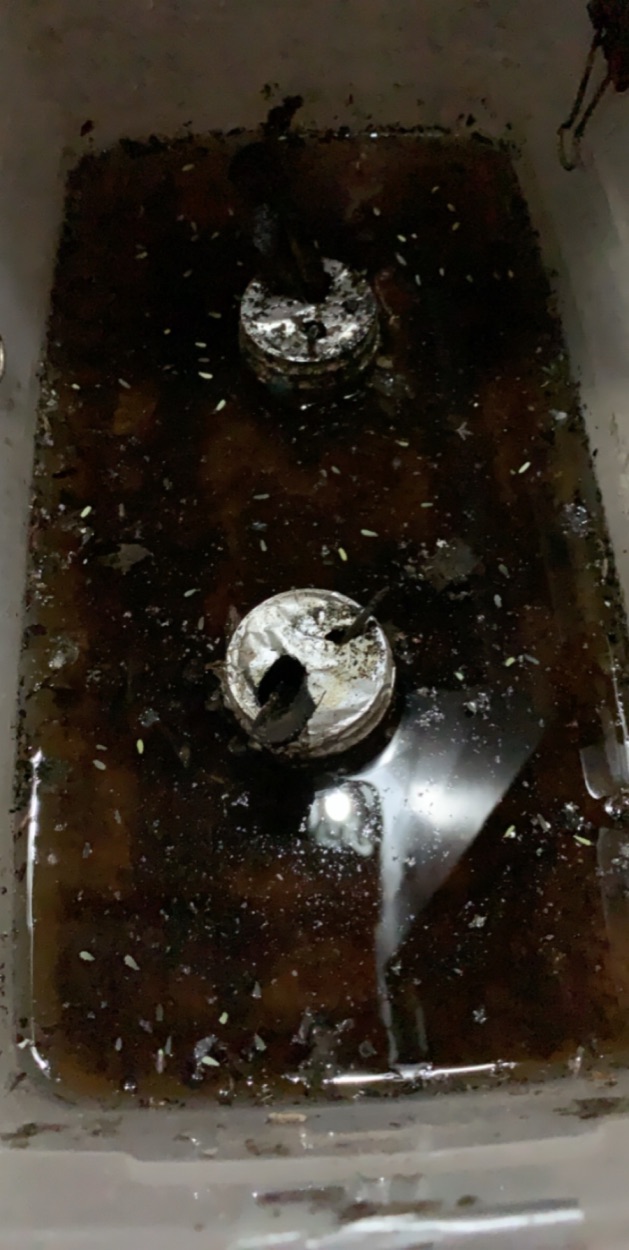
S1a Fig.** An ovipositing trap with undiluted DOM water and ~100 egg rafts recently laid by an adult mosquito in one night. These eggs were retrieved from the trap, hatched in the laboratory, and the larvae were used for the experiments. (Photo Credit: Nicole L Berry)

**S1b Fig.** Solar phototron experimental setup in the field. Note the blue insulation suspending the plastic containers which held the mosquito larvae at the surface waters of the wading pool. One opening in the insulation did not have a plastic container and instead had a garden hose inserted to allow the inflow of cold water throughout the day and maintain temperatures <32°C. (Photo Credit: Nicole L Berry)
